# Patients with III and IV category of the Bethesda System under levothyroxine non-suppressive therapy have a lower rate of thyroid malignancy

**DOI:** 10.1038/s41598-019-44931-8

**Published:** 2019-06-10

**Authors:** Krzysztof Kaliszewski, Dorota Diakowska, Beata Wojtczak, Krzysztof Sutkowski, Bartłomiej Knychalski, Zdzisław Forkasiewicz

**Affiliations:** 1 First Department and Clinic of General, Gastroenterological and Endocrine Surgery, Wroclaw Medical University, Wroclaw, Poland; 2 Department of Nervous System Diseases, Faculty of Health Science, Wroclaw Medical University, Wroclaw, Poland

**Keywords:** Endocrinology, Endocrine system and metabolic diseases, Thyroid diseases

## Abstract

Thyroid nodules (TNs) assigned to the Bethesda System categories III and IV include numerous clinical characteristics, which increase or decrease the risk of malignancy. However, there are very few data regarding the influence of TSH non-suppressive thyroid hormone therapy (NSTHT) on the risk of malignancy in patients in the aforementioned categories. We assessed the number of patients with thyroid nodules assigned to categories III and IV who take TSH NSTHT and if thyroid hormone therapy is associated with a rate of malignancy. We retrospectively analyzed the medical records of 4,716 individuals and selected 532 (11.28%) patients with Bethesda System category III and IV thyroid nodules. All participants underwent surgery, and histopathological verification was obtained in all cases. In all, 33.1% of individuals with category III and IV thyroid nodules took TSH NSTHT. In patients with category III nodules, application of NSTHT was associated with a lower rate of thyroid cancer (TC), though this observation was not significant (OR = 0.55, p = 0.381). In patients with category IV nodules, we demonstrated a significantly lower rate of TC when NSTHT was applied (OR = 0.44, p = 0.005). In conclusion, the prevalence of patients with Bethesda System category III and IV thyroid nodules who take NSTHT is high. TSH NSTHT significantly decreases a rate of malignancy in category IV, but not category III patients.

## Introduction

Since 2009, the Bethesda System for Reporting Thyroid Cytopathology (TBSRTC) has had a well-established role in the diagnosis of thyroid nodules (TNs)^1,2^. Among the six categories in this classification, the third category is known as “atypia of undetermined significance and follicular lesion of undetermined significance” (AUS/FLUS), and the fourth category is known as “follicular neoplasm and suspicious for follicular neoplasm” (FN/SFN)^1,3^. The Bethesda categories III and IV describe varying risks of malignancy. Approximately 5–15% and 10–40% of TNs assigned to AUS/FLUS and FN/SFN categories, respectively, turn out to be malignant on histopathological examination^1^. On one hand, TBSRTC minimizes the number of unnecessary surgeries for thyroid nodules. On the other hand, we cannot estimate the real risk of malignancy associated with the AUS/FLUS and FN/SFN categories because only a minority of these cases undergo surgery. As a result, there is a debate about the best management of category III and IV TNs based on certain clinical characteristics. Currently, various surgical centers have different approaches to treating these lesions^4^, ranging from an observation-only protocol with ultrasound-guided fine-needle aspiration biopsy (UG-FNAB) repeated at six-month intervals to surgery only^5,6^. Because of the great clinical dilemma surrounding the management of thyroid nodules in the AUS/FLUS and FN/SFN categories and the variability in the rates of malignancy in these categories, this subject still garners much discussion. Though the risk of malignancy for category III and IV TNs has been estimated, some authors suggest, that the risk of malignancy for patients with AUS/FLUS and FN/SFN category nodules depends upon the specific clinical situation^3,6^. Others point out that, when using predictive factors for malignancy for the categories of AUS/FLUS and FN/SFN as a risk index, 17% of individuals without the risk factors do not need surgery^3^.

Thus, currently, numerous of clinical characteristics have been described that increase or decrease the risk of malignancy of Bethesda category III and IV nodules. However, there are very few data about TSH non-suppressive thyroid hormone therapy (NSTHT) and its influence on the risk of malignancy in these categories. The next very important issue worthy of closer analysis is the role and impact of thyroid hormone therapy in the management of TNs. Thus, the next question is, how does this therapy influence the risk of malignancy for TNs in the categories of AUS/FLUS and FN/SFN? Currently, it is estimated that, for differentiated thyroid cancers, surgery with subsequent radioiodine therapy followed by thyroid hormone supplementation in suppressive doses is the established treatment procedure.

In addition to the significant and accepted role of levothyroxine (L-T4) in thyroid hormone supplementation, Kantor *et al*. emphasized that L-T4 is one of the most widely and commonly prescribed medications in the United States^7^. Of greater interest, prescriptions for thyroid hormone therapy are steadily increasing for non-supplementary indications^7^. However, to date, the guidelines from 1996 have not been updated and have not recommended the use of thyroid hormone therapy in either suppressive or non-suppressive doses for the treatment of thyroid nodules^8^. These guidelines persist despite cases of modest shrinkage of thyroid nodules observed in patients taking thyroid hormone therapy in suppressive doses^8^. It was estimated that this benefit did not outweigh the potential harm of iatrogenic hyperthyroidism. Despite the American Association of Clinical Endocrinologist and American Thyroid Association Guidelines against the use of thyroid hormone therapy in suppressive doses for the treatment of thyroid nodules, some authors have estimated that almost one-fourth of clinicians prescribe thyroid hormone therapy in non-suppressive doses for thyroid nodules therapy^8^. Because almost 65% of the population have thyroid nodules, this practice may increase the risk of iatrogenic complications in some individuals, especially in the elderly^9,10^. Furthermore, some authors emphasize other disadvantages of L-T4 treatment such as a decrease in bone mineral density, an increase in the risk of atrial fibrillation and other cardiovascular complications^11^.

Many years ago, it was suggested that thyroid hormone therapy in non-suppressive doses reduced or stabilized the size of thyroid nodules^12^. However, this approach to management is still controversial and not accepted by some researchers^9–11^. This hesitancy is in part due to a certain amount of “unpredictable and uncertain” cytological diagnoses of TNs in AUS/FLUS and FN/SFN categories. Currently, it cannot be predicted if TNs assigned to Bethesda System categories III or IV will remain clinically silent or manifest as malignant lesions. Therefore, controversies over the management of these lesions persist. The first question is, “Which nodules assigned to the AUS/FLUS and FN/SFN categories should be considered for surgical treatment and which can be safely observed?” The second question is, “Is thyroid hormone therapy for patients with category III and IV nodules safe? If yes, does the safety extend to both categories?” In our previous study, we presented a description of the clinical features of TNs classified in the AUS/FLUS category and suggested that these lesions had malignant potential. However, we did not investigate the influence of TSH NSTHT on the risk of malignancy.

This study is based on individuals with TNs assigned to the AUS/FLUS and FN/SFN categories, who were taking thyroid hormone therapy in non-suppressive doses and eventually underwent surgery at a tertiary referral center for endocrine surgery. In our department, all patients with FN/SFN category TNs and some selected patients with AUS/FLUS category TNs qualify for surgery.

Therefore, we decided to estimate the number of patients with Bethesda System category III and IV TNs who take L-T4 non-suppressive hormone therapy and how this treatment influences the risk of thyroid malignancy.

## Materials and Methods

Our study protocol was approved by the Bioethics Committee of Wroclaw Medical University (Reference number: KB-783/2017). We obtained oral consent from the participants instead of written consent because the data were analyzed anonymously and retrospectively on the basis of medical records. The process used to obtain oral consent was deemed to be acceptable and was approved by the Bioethics Committee of Wroclaw Medical University. The authors did not have access to any identifying patient information and did not have any direct access to the study participants.

**Figure 1:**
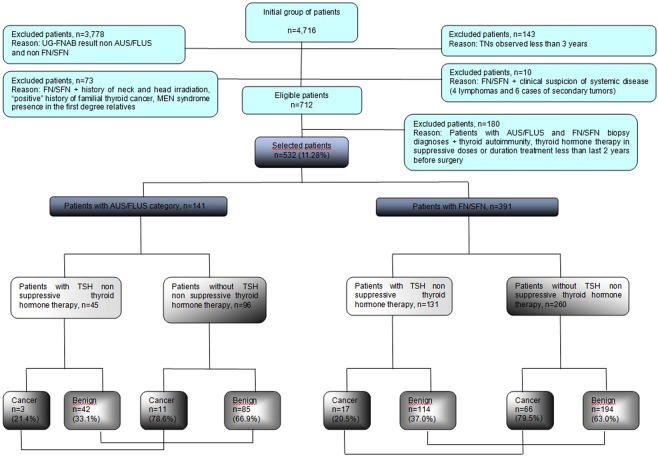
Selection of study group from 4,716 individuals referred for surgery from 2008 to 2017. All participants underwent UG-FNAB before surgery. Histopathological verification was obtained for all participants. UG-FNAB: ultrasound guided fine needle aspiration biopsy, AUS/FLUS: atypia of undetermined significance or follicular lesion of undetermined significance, FN/SFN: follicular neoplasm or suspicious for follicular neoplasm, TNs: thyroid nodules, MEN: multiple endocrine neoplasm, TSH: thyroid stimulating hormone.

The case records of 4,716 patients with thyroid tumors treated consecutively between 1 January 2008 and 31 December 2017 at the Department of General, Gastroenterological and Endocrine Surgery of Wroclaw Medical University (Poland) were analyzed retrospectively. Patients with incidentally detected cancer in a separate TN that was biopsied were excluded from the study. Other exclusion criteria included individuals who had clinical symptoms of malignancy, nodules with dimensions larger than 4 cm, thyroid autoimmunity, previous neck and head radiotherapy and surgery, or family history of thyroid cancer and other thyroid diseases. Autoimmune thyroid disease in patients with FN/SFN and AUS/FLUS was observed in 49 individuals (49/180 additionally excluded; Fig. 1). In this group of patients we observed increased levels of anti-thyroid peroxidase (TPO), anti-thyreoglobulin (Tg), and anti-thyroid-stimulating hormone receptor (TSHR) antibodies. Additionally, autoimmunological process was confirmed in US examination in all of these cases. Of 14 patients with FN/SFN and AUS/FLUS and family history of thyroid cancer (14/73 additionally excluded; Fig. 1) in the first degree relatives we revealed medullary thyroid cancer. Multiple endocrine neoplasia (MEN) syndrome in family history was observed in 6 patients (6/73 additionally excluded; Fig. 1). 53 individuals (53/73 additionally excluded; Fig. 1) had positive history of neck and head irradiation. 10 patients with FN/SFN were excluded due to “other thyroid diseases” such lymphomas (4/10) and secondary tumors (6/10). The inclusion criteria were as follows: the presence of a thyroid nodule or nodules observed for a minimum of 3 years, clearly defined TN features on ultrasonography, euthyreosis, UG-FNAB performed with cytology results confirming AUS/FLUS and FN/SFN categories, and TSH non-suppressive L-T4 therapy conducted at a minimum for the last two years before surgery. The L-T4 doses were adjusted to obtain a serum TSH in range 0.4–4.0 mlU/mL and range 1.12 ± 0.36 µg/kg. Serum TSH, freeT3 and freeT4 levels were measured before surgery and were normal. All patients were operated on by one endocrine surgical team trained in thyroid surgery. The histopathological specimens were analyzed by two pathologists experienced in thyroid diseases. The entire cohort was classified around the time of the surgical treatment under TBSRTC rather than retrospectively reviewed and assigned a category. Only the specimens obtained from UG-FNAB of the thyroid nodules from patients operated in 2008 were retrospectively reanalyzed and assigned to adequate categories according to TBSRTC because this classification was formed and finally recommended in 2009^1^. From the initial group of patients (n = 4,716), 532(11.28%) individuals were selected for further evaluation. Thus, a retrospective analysis of 532 individuals with TNs classified as AUS/FLUS and FN/SFN according to TBSRTC who were taking TSH NSTHT and who underwent surgery was conducted to evaluate an accurate rate of thyroid malignancy rate. There were 437 women and 95 men; the average age was 49.5 ± 15.9 years. The steps for patient selection are presented in Fig. 1.

### Statistical analysis

Descriptive data for qualitative variables are presented as numbers and percentages, and descriptive data for quantitative variables are reported as averages and standard deviations. The distribution of data and homogeneity of variances were tested using Kolmogorov-Smirnov and Levene’s tests, respectively. Quantitative data were compared using Student-t test. Frequencies were analyzed using chi-square test and Fisher exact test. Cochran-Mantel-Haenszel test was used for analysis of stratified categorical data (for two levels of confounding factor). Logistic regression analysis was performed for determination of the impact of thyroid hormone therapy on thyroid cancer occurrence. All tests were two-sided and α ≤ 0.05 was considered statistically significant. Statistical analysis was conducted using Statistica 13.1 software (StatSoft, TIBCO Software Inc., CA, USA).

## Results

The characteristics of the patients in the study group are listed in Table 1. A total of 176(33.1%) of 532(100%) individuals with AUS/FLUS and FN/SFN category TNs took TSH NSTHT. The mean serum TSH levels in patients with NSTHT (176(33.1%)) and without L-T4 therapy (356(66.9%)) were 1.9 mIU/L (range: 0.601–3.93 mIU/L) and 2.1 mIU/L (range: 0.702–4.0 mIU/L), respectively. TSH non-suppressive LT-4 therapy in the first group of patients was administered and conducted at a minimum for the last two years before surgery. The main indication for NSTHT was TN/TNs de novo diagnosis and the opinion of endocrinologists and general practitioners about reducing or stabilizing the growth of thyroid nodules. We did not observed any clinical or biochemical statistically significant differences between these two groups of patients (with NSTHT and without NSTHT). These two groups included to the study differed just only LT-4 supplementation (yes/no). Patients from the total study group were divided into two subgroups according to the final diagnosis. The first group consisted of patients with thyroid cancer (n = 97), and the second group were patients with benign thyroid disease (n = 435). The comparative characteristics of the subgroups of patients with TNs is presented in Table 2. There were no significant differences in gender and age parameters between these two subgroups. However, patients with Bethesda System category IV TNs were represented at a significantly higher rate in the cancer subgroup when compared with patients with benign thyroid disease, and patients with Bethesda System category III TNs were represented at a significantly lower rate in the cancer than in the noncancer subgroup (p = 0.003). The incidence of TSH NSTHT was also significantly lower in the patients with a final diagnosis of thyroid cancer than in patients with benign disease (p = 0.004).

**Table 1:** Baseline characteristics of 532 patients with thyroid nodules with category III + IV cytological diagnosis according to TBSRTC.

Parameters	Number (percent) or average ± SD
**Gender:**	
women	437 (82.1)
men	95 (17.9)
Age (years)	49.5 ± 15.9
**Cytological diagnosis according to TBSRTC:**	
category III	141 (26.5)
category IV	391 (73.5)
**Thyroid hormone therapy**	
yes	176 (33.1)
no	356 (66.9)
**Clinical suspicion of malignancy:**	
no	474 (89.1)
yes	58 (10.9)
**Histopathological diagnosis:**	
struma	227 (42.7)
thyroiditis	19 (3.6)
follicular carcinoma	13 (2.4)
papillary carcinoma	81 (15.2)
medullary carcinoma	4 (0.8)
follicular adenoma	156 (29.3)
Hurtle adenoma	32 (6.0)
**Final diagnosis:**	
cancer	97 (18.2)
noncancer	435 (81.8)

TBSRTC: The Bethesda System for Reporting Thyroid Cytology.

**Table 2:** Relationship between demographic or clinical characteristics and final diagnosis: cancer/noncancer in TN patients. Descriptive data are presented as the number (percent) or average ± standard deviation (±SD).

Parameters	Cancer (n = 97)	Noncancer (n = 435)	p-value
**Gender:**			
woman	83 (85.6)	354 (81.4)	0.33
man	14 (14.4)	81 (18.6)	
Age (years)	50.40 ± 18.11	49.29 ± 15.66	0.542
**Age:**			
<45 year	40 (41.2)	169 (38.9)	0.663
≥45 year	57 (58.8)	266 (61.1)	
**Category of Bethesda:**			
III	14 (14.4)	127 (29.2)	0.003*
IV	83 (85.6)	308 (70.8)	
**Thyroid hormone therapy:**			
yes	20 (20.6)	156 (35.9)	0.004*
no	77 (79.4)	279 (64.1)	

On the basis of data contained in Table 2, Cochran-Mantel-Haenszel analysis of the association between thyroid hormone therapy and the final diagnostic variables was performed, with the parameter of the Bethesda category as a confounding factor. A significant relationship between two binary variables and two levels of confounding factors (Bethesda System categories III and IV) was demonstrated (p = 0.007). This result indicated that an analysis of the association between TSH NSTHT and the risk of malignancy should be performed for category III and for category IV TNs separately. Therefore, the total group of patients (n = 532) was divided into two new subgroups: Bethesda System category III (n = 141) and category IV (n = 391).

Logistic regression analysis for predicting the occurrence of thyroid cancer in association with NSTHT was performed for both subgroups. In the subgroup of patients classified as category III, application of NSTHT decreased the risk of cancer occurrence, though this result was not significant (OR = 0.55, p = 0.381) (Table 3). In the subgroup of patients with Bethesda system category IV TNs, there was a significantly decreased risk of cancer diagnosis when thyroid hormone therapy was applied for the treatment of thyroid lesions (OR = 0.44, p = 0.005) (Table 4).

**Table 3:** Logistic regression analysis of prediction of cancer occurrence (cancer/noncancer, 1/0) in Bethesda System category III nodules. The results were checked by Fisher exact test.

	Cancer (n = 14)	Noncancer (n = 127)	p-value (Fisher test)	Logistic regression analysis		
	N (%)	N (%)		OR	±95%CI	p-value (Wald test)
**Thyroid hormone therapy:**						
yes	3 (21.4)	42 (33.1)	0.287	0.55	0.14–2.11	0.381
no	11 (78.6)	85 (66.9)

**Table 4:** Logistic regression analysis of prediction of cancer occurrence (cancer/noncancer, 1/0) in Bethesda System category IV nodules. The results were confirmed by chi-square test.

	Cancer (n = 83)	Noncancer (n = 308)	p-value (chi-square test)	Logistic regression analysis		
	N (%)	N (%)		OR	±95%CI	p-value (Wald test)
**Thyroid hormone therapy:**						
yes	17 (20.5)	114 (37.0)	0.005	0.44	0.24–0.79	0.005
no	66 (79.5)	194 (63.0)				

## Discussion

The current study included a large single-center cohort of patients with TNs classified as AUS/FLUS and FN/SFN with all individuals undergoing surgery (n = 532). These two categories of TBSRTC are the most controversial cytological groups and are managed completely differently by many departments. This situation exists because of the significant variability in malignancy rates associated with categories III and IV described in the literature^5,13–17^ as well as the significant difference in the percentage of cases with histopathology verification^18,19^. For example, histopathological follow-up in cases of AUS/FLUS range from 30–90% (18%). In the authors’ department, all patients with FN/SFN category TNs and selected individuals with AUS/FLUS category TNs are qualified to surgery. As a result, all patients with category IV and some with category III TNs have histopathological verification. However, in this study, we included only individuals (n = 532, 100%) with AUS/FLUS and FN/SFN category TNs, who had histopathological verification. All patients had UG-FNAB performed a minimum of 1 month to a maximum 6 months before admission and surgical treatment in our department.

Some authors underscore the potential for heterogeneous and subjective interpretation of the specimens assigned to categories III and IV, which could influence subsequent qualification for surgery^14^. Others suggest that the variability in diagnosis is attributable to differences in the populations analyzed, pharmacological management, selection of TN’s and classification bias^1^. The result of these varied opinions is that there is no strict indication for the treatment of thyroid nodules assigned to AUS/FLUS and FN/SFN categories.

In our study 4,716 patients were analyzed with a 100% histopathological follow-up. For patients with nodules classified as AUS/FLUS and FN/SFN and who were treated with TSH NSTHT, we estimated a malignancy rate of 9.92% and 21.22%, respectively. The other important issue that the large group of malignant tumors assigned to Bethesda System categories III and IV turned out to be microcarcinomas. Currently, we know that the oncological potential of these tumors is not clearly established, and the risk of further progression towards aggressive behavior is still uncertain. In such cases, the matter of unnecessary surgeries should be taken into consideration^20^. Additionally, there are very few data about the influence of non-suppressive thyroid hormone therapy on the progression of these lesions.

Currently, it is impossible to predict the potential for malignant evolution of the category III and IV nodules with comparable clinical features. Based on their own observation of the totally independent evolution of two separate nodules in one patient, some authors suggest that individual “intra-nodular factors” are more important for determining progression than the presence or absence of thyroid hormone therapy and clinical and ultrasound characteristics^21^. Sapio *et al*. noticed that the rearrangements of the RET gene in TNs stimulate their growth more rapidly^22^. However, they added, that more studies are needed to use RET rearrangements or other prognostic markers to identify nodules with a predisposition to faster progression. It would be a very helpful diagnostic tool for clinicians to choose the more appropriate therapeutic approach. The other aspect of these hypotheses is the correlation between molecular prognostic markers and thyroid hormone therapy and its influence on the neoplastic progression. We previously described some ultrasound features that are associated with an increase or decrease in the risk of malignancy for AUS/FLUS-classified TNs. However, the controversy still remains. In addition to the association between many clinical characteristics or thyroid hormone therapy with an increase or decrease in the risk of malignancy for category III and IV TNs, some authors have noted that repeat UG-FNAB for initial AUS/FLUS category TNs significantly increases the malignancy rate compared with those without repeated biopsy. It is therefore clear that these authors recommend repeat UG-FNAB for TBSRTC category III nodules on initial biopsy^23^. Other authors suggest additional diagnostic procedures, such as a core needle biopsy or a molecular testing, to be used when “indeterminate cytology” is present^10,24^. In our clinic, all patients classified as FN/SFN qualify for surgery, while selected individuals classified as AUS/FLUS qualify for repeated UG-FNAB six months after the previous biopsy or for surgery. All patients classified as AUS/FLUS included in this study qualified for surgery, and histopathological verification was obtained in all cases. The debatable aspect is the influence of TSH non-suppressive L-T4 therapy on these lesions. Generally, for all thyroid nodules classified as potentially nonmalignant, some authors suggest that in most cases iodine supplementation is sufficient. However, a combination of thyroid hormone therapy and iodine supplementation is considered more efficient for the treatment of larger nodules. Puzziello *et al*. suggest that long-term treatment with L-T4 at a non-TSH suppressive dose significantly reduces their growth^21^. Currently, in the area of Lower Silesian Region (Poland), where all of the participants of our study live, we do not observe any deficiency of iodine in a diet, so no influence on the thyroid malignancy is observed. The main indication for L-T4 non-suppressive therapy for thyroid nodules is its potential role in reducing their size. However, this management approach remains controversial. In our study, we demonstrated a lower rate of thyroid malignancy in patients with thyroid nodules assigned to AUS/FLUS category taking TSH non-suppressive dose of L-T4 compared with patients in the same category, but without thyroid hormone therapy. However, this difference was not significant. Patients with Bethesda System category IV TNs represented a completely different situation. In this group, we found a significant lower rate of thyroid malignancy between the patients who did and did not take thyroid hormone therapy. In the group of individuals with thyroid nodules assigned to FN/SFN taking TSH non-suppressive dose of L-T4 we observed a significantly lower rate of malignancy than the patients without hormonal therapy. The chronic administration of L-T4 at a TSH non-suppressive doses is associated with significantly lower number of malignant tumors in patients with FN/SFN cytology. All analyzed patients assigned to this category had the same clinical and ultrasound features of the biopsied lesions. The two groups of treated and untreated patients were comparable in age, clinical features, initial nodule volume and duration of L-T4 therapy. None had any clinical evidence of an underlying malignant process. No specific parameters predictive of malignancy existed. The main statistically significant parameter in aspect of the occurrence of thyroid malignancy in this group of patients was taking or not NSTHT. However, in the literature there are described clinical and US features increasing the risk of malignancy in FN/SFN nodules like microcalcifications, hypoechogenicity, irregular margins or taller than wide shape measured on a transverse view^5^. Our study demonstrates that patients with thyroid nodules assigned to category IV taking thyroid hormone therapy in non-suppressive doses might be monitored for longer periods of time without surgical treatment than those who are not receiving this therapy. Regarding widespread use of L-T4, we also demonstrated that chronic thyroid hormone therapy in patients with TNs assigned to AUS/FLUS and FN/SFN categories is not associated with a higher rate of thyroid malignancy. Of greater interest, the difference between the number of patients with category IV nodules that were determined to be malignant and that were determined to be benign on final histopathology was higher when the duration of hormonal therapy was longer. We would like to mention, that the difference between the malignancy rates observed in TNs assigned to category III and IV of the Bethesda System may be rather due to small sample size, and not necessarily that NSTHT reduces the risk of malignancy in TNs assigned to category IV, and not to III.

However, our study provides a more accurate correlation of malignancy rates with TNs classified in AUS/FLUS and FN/SFN categories in patients taking thyroid hormone therapy. All analyzed individuals underwent surgery and histopathological verification was obtained in all participants (100%).

In conclusion, our study demonstrates that the prevalence of patients with Bethesda System category III and IV TNs who take thyroid hormone therapy is high. Prolonged treatment with TSH non-suppressive therapy with L-T4 significantly decreases the rate of malignancy in FN/SFN but not in AUS/FLUS category lesions. The important observation is that increasing use of non-suppressive L-T4 therapy in the management of TNs does not enhance the rate of thyroid malignancy.

## Data Availability

The datasets analysed during the current study are available from the corresponding author on reasonable request.
